# Postmortem Metabolomics of Insulin Intoxications and the Potential Application to Find Hypoglycemia-Related Deaths

**DOI:** 10.3390/metabo13010005

**Published:** 2022-12-20

**Authors:** Liam J. Ward, Gustav Engvall, Henrik Green, Fredrik C. Kugelberg, Carl Söderberg, Albert Elmsjö

**Affiliations:** 1 Department of Forensic Genetics and Forensic Toxicology, National Board of Forensic Medicine, 587 58 Linköping, Sweden; 2 Department of Forensic Medicine, National Board of Forensic Medicine, 587 58 Linköping, Sweden; 3 Division of Clinical Chemistry and Pharmacology, Department of Biomedical and Clinical Sciences, Linköping University, 581 83 Linköping, Sweden

**Keywords:** acylcarnitines, biomarkers, death investigation, forensic science, hyperglycemia, hypoglycemia, insulin, metabolomics, multivariate statistics

## Abstract

Postmortem metabolomics can assist death investigations by characterizing metabolic fingerprints differentiating causes of death. Hypoglycemia-related deaths, including insulin intoxications, are difficult to identify and, thus, presumably underdiagnosed. This investigation aims to differentiate insulin intoxication deaths by metabolomics, and identify a metabolic fingerprint to screen for unknown hypoglycemia-related deaths. Ultra-high-performance liquid chromatography-quadrupole time-of-flight mass spectrometry data were obtained from 19 insulin intoxications (hypo), 19 diabetic comas (hyper), and 38 hangings (control). Screening for potentially unknown hypoglycemia-related deaths was performed using 776 random postmortem cases. Data were processed using XCMS and SIMCA. Multivariate modeling revealed group separations between hypo, hyper, and control groups. A metabolic fingerprint for the hypo group was identified, and analyses revealed significant decreases in 12 acylcarnitines, including nine hydroxylated-acylcarnitines. Screening of random postmortem cases identified 46 cases (5.9%) as potentially hypoglycemia-related, including six with unknown causes of death. Autopsy report review revealed plausible hypoglycemia-cause for five unknown cases. Additionally, two diabetic cases were found, with a metformin intoxication and a suspicious but unverified insulin intoxication, respectively. Further studies are required to expand on the potential of postmortem metabolomics as a tool in hypoglycemia-related death investigations, and the future application of screening for potential insulin intoxications.

## 1. Introduction

Metabolomics is defined as the comprehensive and quantitative analysis of all metabolites within a specified biological system under study, the metabolome [1]. Untargeted metabolomics has seldom been utilized in the postmortem context in humans. Previously, only a few studies have investigated the associations between metabolome differences and postmortem interval (PMI) [2,3,4]. However, advances have been made recently in expanding the utility of untargeted metabolomics as a potential tool in death investigations, specifically in deaths related to pneumonia [5] and oxycodone intoxication [6]. In these studies, untargeted metabolomics using multivariate statistical modeling could classify specific causes of death, with high sensitivity and specificity, based on the metabolic fingerprint of postmortem blood samples [5,6]. In addition, metabolomics has been used within a forensic context to investigate cause of death using cerebrospinal fluid [7], in a case employing urinary postmortem metabolomics [8], as well as postmortem redistribution mechanisms [9]. This highlights the applicability of postmortem metabolomics in forensic death investigations.

Hypoglycemia-related deaths can result from various causes, with the most common deriving from excessive alcohol consumption, the effects of malnutrition and/or prolonged fasting, and organ failure—particularly the liver and kidneys [10]. However, a cause of particular interest to forensic investigations is hypoglycemic death as a result of insulin intoxication, as these are difficult to diagnose postmortem. Death by insulin intoxication is relatively rare, with most cases arising from an accidental overdose, suicide, and homicide cases [10]. However, the postmortem determination of insulins in human biological matrices is challenging where cellular degradation and hemolysis are apparent [11]. Therefore, insulin intoxications are difficult to diagnose without the support of, preferentially, antemortem blood samples with the analyses of insulin and C-peptide levels, or circumstantial paraphernalia (insulin vials, syringes, etc.) present at the scene of death. Therefore, it is presumed that the real number of insulin intoxication-related deaths is underrepresented [10,12], highlighting a real issue in need to further study within the field of forensics.

Metabolomics seems to be a logical methodology for the investigation of insulin intoxications, as insulins directly affect glucose metabolism within humans. Moreover, metabolomics has been utilized in numerous studies to investigate insulin resistance and diabetes demonstrating significant alterations to the metabolome [13,14,15]. Thus, this investigation aims to assess if postmortem metabolomics could be used to identify differences in the metabolome between confirmed insulin intoxication deaths and two control groups—a hyperglycemic and normoglycemic group. The hypothesis is that significant differences in the metabolome will be able to distinguish the different glycemic states from one another. In addition, this study aims to assess if a postmortem metabolomics screening tool could be built, using the identified metabolic fingerprint for insulin intoxication, and use it to help identify previously unknown hypoglycemic-related deaths.

## 2. Materials and Methods

### 2.1. Study Population

Autopsy cases admitted to the National Board of Forensic Medicine, Sweden, between July 2017 and November 2020 were considered for inclusion in the study (*n* = 17,011). This includes autopsy cases from all six regional Departments of Forensic Medicine across Sweden, with all samples for toxicological screening being transported from each regional site to the Department of Forensic Genetics and Forensic Toxicology, Linköping, Sweden. The causes of death, primary and/or contributing, were screened for cases related to insulin intoxication, which were to be included in the study group of deaths attributed to hypoglycemia (group name “hypo”). These causes of death descriptions, translated from Swedish, included “insulin overdose”, “(suspected) drug poisoning with insulin”, “insulin coma from insulin poisoning”, and “hypoglycemic coma from exogenous insulin”. To maximize the total number of insulin intoxication cases available, drug poisoning with multiple substances that included insulin was considered. It should be noted that these insulin intoxication cases are referred to as confirmed within this study. This is based on the forensic pathologists’ diagnosis that the cause of death included insulin intoxication. Due to the inherent difficulties in insulin analyses postmortem, this diagnosis is not necessarily confirmation that insulin has been positively identified in postmortem biological samples, but it may also be confirmed as a result of strong circumstantial evidence. Due to the large pool of samples that had undergone postmortem toxicological screening using femoral blood, and this matrix is considered the golden standard for intoxication determination in toxicological screening [16,17], this was selected as the biological matrix of study. Exclusion criteria for the hypo group included lack of femoral blood sample with corresponding postmortem toxicological screening (*n* = 2), and hospitalization during the antemortem period (*n* = 2) to remove any bias from the medical treatment provided during this period. Thus, the hypo group included a total of 19 cases.

Two additional study groups were considered for this investigation, a negative-study group that consisted of cases with hyperglycemic deaths (group name “hyper”) with the primary cause of death diagnosed as “diabetic coma”, and a neutral-study group presumed to be normoglycemic (group name “control”) with a solitary cause of death by “hanging”. For the hyper group, the inclusion criteria were a postmortem vitreous humor (VH) glucose level > 10.0 mmol/L confirming hyperglycemia [18].

Table 1 presents the demographic overview of the cases included in this study. The study groups were matched at a ratio of 1:1:2, resulting in *n* = 19, *n* = 19, and *n* = 38 cases for the hypo, hyper, and control groups, respectively. These study groups were matched to the hypo group in relation to age, sex, and body mass index (BMI). The PMI window used in this investigation was decided by the cases included in the hypo group; thus, to reduce the impact of differing PMI windows between groups, PMI was controlled between groups so that there were no significant differences. VH glucose levels were more than 10.0 mmol/L for all cases included in the hyper group. None of the hypo cases had VH glucose levels greater than 10.0 mmol/L, although two cases had no available data. VH glucose analysis was not performed for those cases included in the control group.

Positive drug and/or substance use found in postmortem toxicological screening for cases included in all groups can be found in Supplement Table S2. Whereas cases included in the hypo group included those with poly-drug intoxications, the cases in the hyper and control groups were included on the basis that they had no other contributing causes of death. Thus, positive drug/substance use found in toxicological screening does not constitute toxic levels, nor a contributing cause of death.

### 2.2. Test Population

A test group of cases was collated to assess if the metabolomics model could be used as a screening tool to identify previously unknown death attributable to hypoglycemia. To mimic a real-world application of such a model, the test group included a pseudo-random selection of autopsy cases selected from the inclusion period. The only criterion for this was to have a similar sex distribution. Thus, the first 10 male and 10 female cases were selected for each month within the inclusion period, resulting in the selection of 820 cases (410 males and 410 females). This group was reduced to 776 cases (390 males and 386 females) after the exclusion of cases under the age of 18 (*n* = 12), cases with no available toxicological screening data (*n* = 28), and any cases that were previously included in any of the previous study group described—hypo, hyper, or control (*n* = 4). Table 2 presents a demographic overview of the test group.

### 2.3. Postmortem Blood Screening

Postmortem femoral blood samples were analyzed using the ultra-high-performance liquid chromatography-quadrupole time-of-flight mass spectrometry system (UHPLC-QTOF; Aligent 1290 Infinity LC with Agilent 6540 QTOF, Agilent Technologies Sweden AB, Stockholm, Sweden) with standardized procedures previously described [19]. Briefly, samples were prepared via protein precipitation, the addition of three internal (amphetamine-d8, diazepam-d5, and mianserin-d3), and injected into the UHPLC-QTOF system. Separation was performed via a gradient elution on a C18 column (150 mm × 2.1 mm, 1.8 µm; Waters Acquity HSS T2 column, Waters Sverige AB, Stockholm, Sweden), followed by mass spectrometry (MS) acquisition in positive mode for a total run time of 12 min. Each analytical run included a blank whole blood sample, with internal standards, run at the beginning and end of each run. This method is a routine method for the analyses of medicines and drugs possible to be analyzed via UHPLC-QTOF in positive mode.

Mass spectra were processed using XCMS and CAMERA packages in R (v.4.1.2) for peak list generation and peak annotation, respectively. The parameters used for XCMS peak processing are included in Supplement Table S1, and the associated R code is included in Supplement File S1.

### 2.4. Multivariate Modeling and Statistical Analyses

All chromatographic peaks with a retention time of <90 s and >660 s were excluded from the analysis. The data were normalized using probabilistic quotient normalization, using a reference spectrum calculated from the median [M]+ peaks across the control group samples.

The hypo, hyper, and control groups, totaling 76 postmortem cases, were used for multivariate modeling using SIMCA v.17 (Sartorius AG, Göttingen, Germany). The normalized data were unit variance (UV)-scaled and log-transformed. Orthogonal partial least squares discriminant (OPLS-DA) was used for feature selection, with two models comparing hypo vs. control groups and hypo vs. hyper groups. Q2 and R2 values were used to evaluate the prediction capability of the model and how well the model explains the dataset, respectively, and analysis of variance testing of the cross-validated predictive residual (CV-ANOVA) was used to evaluate the reliability of the model, with a *p*-value < 0.05 considered significant. Features with a variable influence on projection (VIP) values > 1.0 with a lower 95% confidence interval > 0, in both OPLS-DA models, were selected for modeling on a shared-and-unique structures (SUS) plot. In the SUS plot, features that correlated along the diagonal, beyond the p(corr) thresholds of −0.2 and 0.2, were considered uniquely discriminant of the hypo group.

Univariate statistical analyses were performed by Student’s *t*-*test*, with posthoc Benjamini–Hochberg FDR adjustment using Excel 2019 (Microsoft, Redmond WA, USA).

### 2.5. Metabolite Identification

Metabolite identification was performed by matching the unknown feature mass-to-charge (*m*/*z*) ratio to online public databases, the human metabolome database (HMDB), and the METLIN database. In accordance with the Metabolomics Standards Initiative (MSI) guideline for feature annotation, this public database matching corresponds to level 2 or 3 putative metabolite identification [20].

### 2.6. Postmortem Metabolomics Screening Using a Class Prediction Model

Identified metabolites and unidentified features that discriminated the hypo group in the SUS plot were used for the postmortem metabolomics screening test, via the use of the class prediction function in SIMCA. This method is aimed to assess if postmortem metabolomics can be used to filter out (“screen”) any potential cases that may be considered hypoglycemia-related. Herein, a three-group OPLS-DA model was built using only these features, and then the test group of random autopsy cases was loaded into the model for class prediction using a classification table. A positive class prediction was given for those cases that achieved a probability score of >0.65 with one or more of the model-building classes—hypo, hyper, and/or control. Additional review, implemented after the initial screening, included collating the diagnosed causes of death for cases screened to each group, as well as a review of any available VH glucose values (specific for identifying hyperglycemic cases only). Specifically, for the cases screened to hypo, any case that had an undetectable cause of death was subject to an in-depth autopsy case review to establish if any potential hypoglycemia-related causes were present.

## 3. Results

A demographic overview of the cases selected for the three main study groups—hypo, hyper, and control—is included in Table 1. As the groups were matched for sex, age, BMI, and controlled for PMI during inclusion, there were no significant differences observed. In addition, the toxicological screening was reviewed, and Supplement Table S2 describes the frequencies of positive drug and alcohol findings within the postmortem samples for hypo, hyper, and control groups. Of important note, the positive drug and alcohol use described for the hyper and control cases was independent of any diagnosed causes of death by the forensic pathologists, and likely present therapeutic drug use and normal consumption of alcohol.

**Figure 1:**
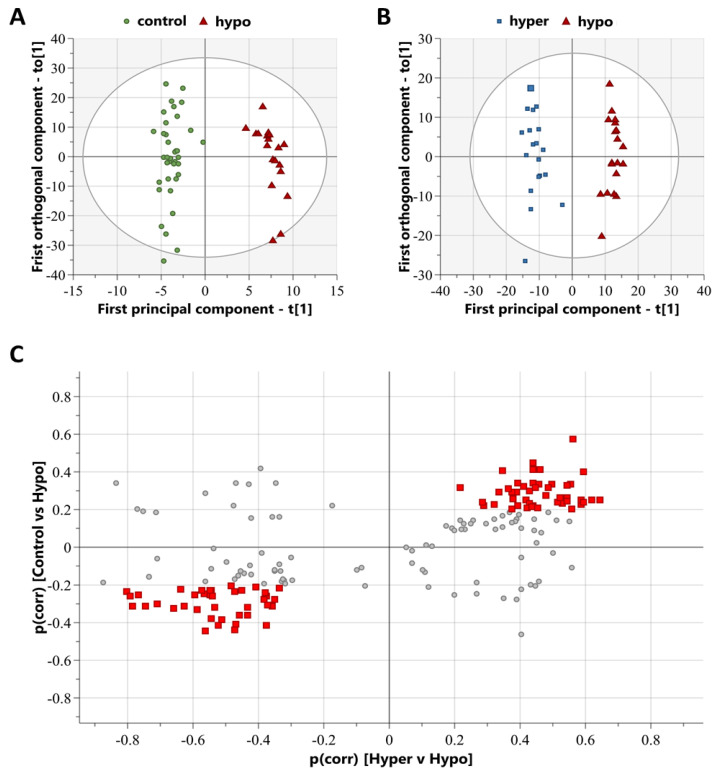
Multivariate modeling can be used to distinguish study groups and highlight metabolite features discriminating the hypo group. (**A**) Orthogonal partial least squares discriminant analysis (OPLS-DA) score plot of the model comparing control (green circles) and hypo (red triangles) groups, R2 = 0.96, Q2 = 0.72, CV-ANOVA < 0.001. (**B**) OPLS score plot of the model comparing hyper (blue squares) and hypo (red triangles) groups, R2 = 0.96 and Q2 = 0.86. (**C**) Shared-and-unique structures (SUS) plot highlighting metabolite features (red squares) that discriminate the hypo group from the hyper and control groups. SUS plot was built from the two OPLS-DA plots, with control vs. hypo on the *y*-axis, and hyper vs. hypo on the *x*-axis. Hypo group discriminating features (red squares) distribute along the diagonal, with the threshold set as p(corr) < −0.2 and p(corr) > 0.2. Non-discriminating features are those plotted as grey circles.

Mass spectra from the hypo, hyper, and control cases were processed using XCMS to compile a comprehensive list of chromatographic peaks with specific accurate masses and retention times, termed features. After the exclusion of features with a retention time of <90 s and >660 s, this selection results in 2314 features being available for multivariate modeling. Multivariate modeling using unsupervised principal component analysis (PCA) score plots revealed a group overlap between control and hypo groups (Supplement Figure S1A) and respective group clustering between hypo and hyper groups (Supplement Figure S1B). The PCA score plot between hypo and hyper revealed two outliers within the hypo group; however, a review of the case information and mass spectra runs could not determine any distinct reasoning for this, and they were not outliers in the PCA score plot for hypo and control; therefore, they were retained for future analyses. Multivariate modeling using supervised OPLS-DA analyses was successful in distinguishing study groups by metabolic features. The hypo vs. control OPLS-DA model resulted in a significant model with R2 = 0.96 and Q2 = 0.72, with a CV-ANOVA *p*-value < 0.001 (Figure 1A), and the hypo vs. hyper OPLS-DA model resulted in a significant model with R2 = 0.96 and Q2 = 0.86, with a CV-ANOVA *p*-value < 0.001 (Figure 1B). Combining these two OPLS-DA models into a SUS plot resulted in 85 features that specifically discriminated the hypo group (Figure 1C). These discriminating features include those that are either increased or decreased in abundance in the hypo group, compared to the control and hyper groups, which distribute to the top-right and bottom-left of the SUS plot, respectively (Figure 1C).

As confirmation, additional analyses were performed using the same cases as above, but only including data for those 85 features that were discriminant of the hypo group in the SUS plot. Clear group separation was present in the OPLS-DA models comparing hypo vs. control (R2 = 0.93, Q2 = 0.73, CV-ANOVA < 0.001; Supplement Figure S2A) and hypo vs. hyper (R2 = 0.89, Q2 = 0.75, CV-ANOVA < 0.001; Supplement Figure S2B).

Online public database matching for the identification of the 85 features discriminating the hypo group resulted in putative metabolite identifications metabolites, listed in Table 3. One class of metabolites was highlighted with 13 acylcarnitines identified, all at reduced levels in the hypo group compared to both control and hyper groups. Univariate statistical analysis confirmed that 12 acylcarnitines were significantly reduced in the hypo group compared to the hyper group (Table 3). The remaining eight identified metabolites belonged to various metabolite classes. Of these, four were significantly increased in the hypo group compared to the hyper group (Table 3). Discriminating metabolite features that could not be identified via database matching are listed in Supplement Table S3.

**Figure 2:**
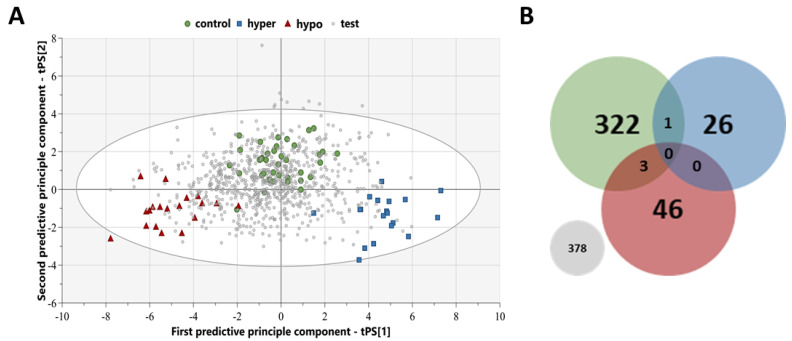
Screening, using a class prediction model, of the random test group of postmortem cases. (**A**) Orthogonal partial least square-discriminant analysis (OPLS-DA) score plot of the model built only including metabolic features from the shared-and-unique structures (SUS) plot that discriminant the hypo group (red triangles) from the hyper (blue squares) and control (green circles) groups. The random test cases (grey diamonds) were then screened using this class prediction model, built using the 85 metabolic feature discriminating insulin intoxications, and assigned group membership once the probability threshold of 0.65 was surpassed. (**B**) Venn diagram of the classification model with the number of test cases predicted to belong to each group: hypo (red), hyper (blue), control (green), and no group (grey).

The three-group OPLS-DA model, only using the features that discriminated the hypo group, resulted in clear group separation (R2 = 0.76, Q2 = 0.46, CV-ANOVA < 0.001; Figure 2A). For the screening analysis via class prediction, a randomly selected test group of cases (*n* = 776) was selected from the inclusion period, with demographics presented in Table 2. The screening resulted in 46 cases screened to hypo, 26 cases screened to hyper, 322 cases screened to control, 3 cases screened as both hypo and control, 1 case screened as both hyper and control, and the remaining 378 cases not surpassing the probability threshold of 0.65 required for class prediction (Figure 2B).

Those test group cases that were screened as hypo (*n* = 46) underwent subsequent review of the cause of death. Table 4 lists the groups of primary causes of death that were attributed to this subset, with twenty cases being cardiovascular-related, eight cases as pulmonary-related, six cases of substance overdose/poisoning, two cases each of traumatic head injury and liver cirrhosis, one case each of acidosis and starvation, and six cases of an undetectable cause of death. VH glucose levels were also reviewed, where available (*n* = 23), to identify any cases that were hyperglycemic VH glucose levels > 10.0 mmol/L. Only two cases had VH glucose levels indicating hyperglycemia, one cardiovascular complications case and one substance overdose/poisoning case; all other cases with available data were not hyperglycemic.

Individual autopsy reports were reviewed, by a forensic pathologist, for the acidosis case, as diabetes was listed as a secondary contributing factor to the cause of death, and all six cases of an undetectable cause of death. This review resulted in the acidosis case being an individual with metformin intoxication as a secondary contributing factor to the cause of death. In addition, one of the unknown cases was an individual with diabetes mellitus type 1 where insulin analysis was requested by the forensic pathologist at the time of autopsy; however, sample quality impeded the analysis of insulin. Three unknown cases were noted to suffer from alcoholism, one case was noted to have lost a substantial amount of weight prior to death with the effects of starvation possibly contributing, and the remaining unknown case had no relevant notes pertaining to a possible cause of hypoglycemia.

As metabolic differences between the hypo and hyper groups showed significant differences, the test cases screened to the hyper group were also reviewed for cause of death (Table 5). This was to observe if any test cases screened as hyper contained any hyperglycemic-related causes of death. Primary causes of death attributed to test cases screened as hyper included eight substance overdose/poisonings, seven diabetes mellitus-related, three each of cardiovascular-related and hangings, one each of multiple organ failure, burns/inhalation of smoke and ketoacidosis, and two undetectable causes of death. VH glucose levels were also reviewed, where available (*n* = 13), and it was found that seven cases had VH glucose levels > 10.0 mmol/L indicating hyperglycemia, six of which were those with diabetes mellitus complications as a cause of death and one substance overdose/poisoning. Moreover, the seven diabetes mellitus-related cases represented 54% of the total causes of death attributed to diabetes mellitus complication in the complete test group, the remaining cases being classified as either control (*n* = 1) or no class (*n* = 5).

In addition, the test cases screened as control, no class, and multiple classes are presented in Supplement Table S4–S6, respectively.

## 4. Discussion

The identification of deaths attributable to hypoglycemia, particularly those resulting from insulin intoxication, is currently a challenge during death investigations. In the current study, we have used postmortem metabolomics as a tool in the screening of possible hypoglycemia-related deaths. Distinct differences in the metabolome between hypo, hyper, and control groups were evident, allowing for a unique hypo metabolic fingerprint to be used to help identify previously unknown deaths that may have contributing hypoglycemic components.

Postmortem metabolomics has been suggested as a tool in death investigation, with prior publications demonstrating the applicability in discriminating deaths attributable to pneumonia [5] and oxycodone intoxication [6]. These previous studies demonstrated the robustness of combining postmortem metabolomics and multivariate modeling, with validation testing resulting in sensitivity and specificity values between 80–86% and 76–84% [5,6]. Unfortunately, in the current investigation, the sensitivity and specificity of the model were not possible to validate, due to only having 19 insulin intoxication cases available that were needed to build the multivariate model. However, strong group separation was clearly evident with significant cross-validation within the individual OPLS-DA models discriminating hypo and control, hypo, and hyper, and even the three-group hypo, hyper, and control model. This strong separation and significant cross-validation within the modeling provides confidence that differences are present between the metabolomes of the different study groups.

### 4.1. Acylcarnitine Profile as a Potential Marker for the Glycemic Condition in Postmortem Cases

Acylcarnitines were found at reduced levels in the hypo group, with significant reduction for almost all identified acylcarnitines comparing hypo and hyper groups. Acylcarnitines are a large group of metabolites, with the main function of transporting fatty acids to the mitochondria for beta-oxidation [21]. An increasing number of diseases and conditions are presenting with association with distinct acylcarnitine profiles, including cardiovascular disease [22,23,24], and recently highlighted using postmortem metabolomics oxycodone intoxications [6]. Interestingly, in this previous postmortem metabolomics study, a significant reduction in acylcarnitines was observed when comparing oxycodone intoxications and non-intoxication groups [6]. A comparison of the identified acylcarnitines revealed four common acylcarnitines in the present study—heptanoylcarnitine (C7), dodecenoylcarnitine (C12), hydroxyhexadecenoylcarnitine (C16:1-OH), and hydroxyhexadecanoylcarnitine (C16-OH). This overlap in acylcarnitines could be indicative of similar mechanisms occurring in the agonal period prior death between oxycodone and insulin intoxications. However, as the classification screening shows substance overdose/poisonings in all classification groups, the least classified as hypo (0.8%), followed by hyper (1.0%), control (6.2%), and no class (10.0%), the overlap may just be coincidental. Additional screening into the causes of substance overdose/poisonings, together with further investigation, would be required to assess any similar intoxication related mechanisms related to these specific acylcarnitines.

Insulin resistance and glucose metabolism have also been implicated with distinct acylcarnitine profiles [21]. Both medium- and long-chain acylcarnitines, with ester carbon chain lengths ranging from C6-C12 and C13-C20, respectively, have been shown to play a role in insulin resistance, glucose metabolism, and diabetes risk [25,26,27].

Long-chain acylcarnitines are emerging as important metabolites in energy metabolism [21]. Long-chain acylcarnitines, as intermediates of fatty acid metabolism, were proposed as a potential marker for insulin resistance [28]. However, later, it was reviewed that long-chain acylcarnitines are active intermediates that may play a role in the development of insulin resistance [29]. This was further demonstrated in cell and ex vivo models with altered glucose metabolism via long-chain acylcarnitine-induced inhibition of pyruvate and lactate oxidation in mitochondria [27,30]. All of the long-chain acylcarnitines observed in this study were hydroxylated, and in a previous diabetic murine model, hydroxylated-acylcarnitines (including those found here: C14:1-OH, C16-OH, C16:1-OH, C16:2-OH, C18-1-OH) were found increased in the diabetic heart compared to control heart, and this was theorized to cause mitochondrial injury, reducing energy production and increasing oxidative stress [31]. In the current study, these hydroxylated-acylcarnitines are found at significantly reduced levels in the hypo group compared to the hyper group; this may implicate a role of insulin-signaling in hydroxylated-acylcarnitine production. The interaction between insulin-signaling and long-chain acylcarnitine production is of particular interest. The heart muscle and skeletal muscle are the main sources of long-chain acylcarnitines [32,33], and the activation of insulin-signaling decreases the carnitine palmitoyl transferase-I activity reducing the production of long-chain acylcarnitines [34]. Moreover, it has been hypothesized that long-chain acylcarnitines inhibit glucose uptake and metabolism to reduce the risk of hypoglycemia [30]. Thus, increased insulin signaling reduces the production of long-chain acylcarnitines; therefore, allowing glucose uptake and metabolism and increasing the risk of hypoglycemia. Interestingly, the pharmacological administration of a single dose of palmitoylcarnitine, a long-chain acylcarnitine, inhibited insulin signaling and insulin-dependent glucose uptake in a murine model [35]. This may indicate that the insulin/acylcarnitine interplay may be dependent on the prevalent concentrations of one or the other.

Medium-chain acylcarnitines have been implicated in diabetes, commonly found at elevated blood concentrations [21], including both the C8-OH [36] and C12 acylcarnitines [37,38] that were identified in the current study. Medium-chain acylcarnitines, specifically the C10 and C12 acylcarnitines, have been proposed as possible contributors to insulin resistance [38]; however, the mechanisms behind the interactions of these medium-chain acylcarnitines and insulin signaling are unclear. If there are similar mechanisms to long-chain acylcarnitines, then the administration of insulin may also reduce the production of medium-chain acylcarnitines.

Of the three short-chain acylcarnitines identified in the present study, C4-OH has been previously reported to be associated with insulin resistance and type 2 diabetes mellitus [39]. Moreover, C4-OH is primarily generated from the corresponding ketone body—beta-hydroxybutyrate [40], which has been evaluated for its potential in identifying ketoacidosis in postmortem cases [41]. The study by Ahlström et al. [41] and the current study have an overlap in cohorts; however, too few test cases classified as potentially hypo or hyper had beta-hydroxybutyrate analyses performed, presumably due to the lack of acidosis suspicion regarding the various causes of death.

In the present study, C4-OH, three medium-chain acylcarnitines, and six long-chain acylcarnitines show significant reductions in the hypo group of insulin intoxication cases, compared to the hyper group of diabetic coma cases, which could be indicative of the effects of insulin signaling and acylcarnitine interplay. Non-significant reductions were observed for all acylcarnitines when comparing the hypo group and control group, which are presumed to be normoglycemic, and this may be due to a lack of power in this comparison with the limited number of insulin intoxication available. Thus, the acylcarnitine profile may be of use in the postmortem setting as a marker of the glycemic state in deceased individuals, in which a decreased acylcarnitine profile is indicative of hypoglycemia and an increased profile is indicative of hyperglycemia. However, further studies are warranted to assess the acylcarnitine comparison between hypo-/hyperglycemic and normoglycemic cases with a greater number of autopsy cases, particularly hypoglycemic cases.

### 4.2. Other Metabolites Discriminant of the Insulin Intoxication Group

Four other metabolites, discriminant of the hypo group, were all found at significantly increased levels in the hypo group compared to the hyper group, with a non-significant increase compared to the control group.

7-methylguanine, a purine metabolite, has been positively associated with incident type 2 diabetes mellitus independent of traditional risk factors [42]. Although, in a lifestyle intervention study of prediabetic men, 7-methylguanine was associated with regression toward normoglycemia [43]. Further studies are required to elucidate the potential mechanisms between 7-methylguanine and phosphatidylcholines with insulin signaling and glucose metabolism.

3,5-dihydroxyphenylvaleic acid and sphinganine 1-phosphate have no evident association with insulin signaling or glucose metabolism within the current literature.

### 4.3. Strengths and Limitations of Postmortem Metabolomics and Metabolic Fingerprinting

A common strength and weakness of the current study is the number of confirmed insulin intoxication used. Within the forensic toxicology field, a research investigation involving data from 19 confirmed insulin intoxication cases is extremely high, as the majority of insulin intoxications are presented as case studies as confirmed cases present so infrequently. It must be repeated that though termed “confirmed” insulin intoxications are used, this is based on the assumed correct cause of death diagnosis given by the forensic pathologist; this diagnosis can be given due to a verified insulin analysis, but also by strong circumstantial evidence associated with the death. However, this number of confirmed cases limited our capabilities in being able to withhold a proportion of the cases for model validation and building a model with a training and a test set of data.

The control group used in this investigation was selected from a pool of hanging cases, which had no other contributing causes of death recorded. Though it is evident that hanging as a cause of death also affects the metabolome, as demonstrated in proof-of-concept animal models of mechanical asphyxiation [44], we believed this group was a suitable control group as they represent a varied postmortem group comprising both sexes, a variety of ages, and BMI—all factors we wished to control for in the study group matching during study design. Additionally, it should be noted that hanging is a common suicide method; thus, many of these cases will have had psychiatric comorbidities. Several control cases, alongside both hypo and hyper cases, were positive for drug use in postmortem toxicological screening, including antidepressants and anxiety medication (Supplement Table S2). However, for the hyper and control cases, these drug and alcohol usages were deemed independent of diagnosed causes of death by the forensic pathologists. For the hypo group, due to the need to retain as many of these cases as possible poly-drug intoxications were included in this group as described in the methods section. Though not a perfect control group, something that is exceedingly difficult to establish in human postmortem cases, we believe hanging constitutes the best fit for a normal postmortem population in the forensic setting.

Additionally, another limitation of the control group was that they were assumed to be normoglycemic. This assumption is without any confirmatory analyses of glucose levels. However, it is known that the glycemic condition is particularly difficult to establish in postmortem samples, and the most commonly used method of VH glucose measurements is only suitable for assessing if hyperglycemia is present, with values exceeding 10.0 mmol/L [18]. This VH glucose threshold was employed as an inclusion criterion for the hyper group to ensure all cases were hyperglycemic, and also controlled for in the hypo group to ensure none were hyperglycemic. However, a minor limitation should be noted that VH glucose measures were not available for two of the hypo cases.

As we had a limited number of confirmed insulin intoxication cases, we could not employ an inclusion criterion on a specific PMI window; thus, the hypo group includes cases with a broad PMI window (with a quartile range 4–10 days); this is a major limitation of the study as PMI affects the metabolome of various biofluids and tissues [45]. However, we have minimized this bias as much as feasibly possible by controlling that there are no significant differences in the PMI widow between the model-building groups (hypo, hyper, and control). Aside from the PMI window limitation, the group matching in relation to sex, age, and BMI is a particular strength of the study design given that postmortem samples are very heterogeneous, and this group matching limits the potential bias originating from such sources. Future metabolomic investigations should investigate the differences such as demographic variables have on the postmortem metabolome, and in particular, a much-needed effort is required to fully elucidate the effects of PMI on the human metabolome.

A key limitation of the OPLS modeling is that it is a supervised method; thus, the model intrinsically assumes that the group classification for the cases used to build the model is perfect. Another major limitation is that though the diagnostic accuracy of cause-of-death determinations has not been reported, clinical diagnostic accuracy has reported a potential error rate of 10–15% [46]. However, in the current study, we also assumed that the forensic pathologists’ diagnoses for the cause of death were correct.

### 4.4. Postmortem Metabolomic Screening as a Potential Tool for Aiding Cause of Death Investigation

A screening application was developed for testing our hypo postmortem metabolomics model by conducting a class prediction model on a random selection of 776 autopsy cases for any previously unknown deaths with a potential hypoglycemic cause. This screening resulted in a discreet number of cases, only 5.9%, as potentially hypoglycemia related. A review of VH glucose levels identified two cases that had hyperglycemic VH glucose levels. Additionally, the review of diagnosed causes of deaths revealed that the majority of cases screened as hypo had a diagnosed cause of death. Six cases of undetectable causes of death, as well as one exceptional case of ketoacidosis with diabetes mellitus provided as a contributing cause of death, were then considered as potential true-positives for in-depth review of autopsy case review. A review of the autopsy case reports revealed three individuals as suffering from alcoholism; thus, a motive for hypoglycemia could be excessive alcohol consumption before death. One individual had notes regarding a substantial weight loss before death; thus, hypoglycemia because of starvation could be motivated. Finally, two individuals had diabetes mellitus diagnoses. One case was a type 2 diabetic, with acidosis listed as a primary cause of death and metformin intoxication listed as a secondary contributing cause of death. Metformin is an antidiabetic agent that reduces blood sugar levels; thus, a clear motive for hypoglycemia was present. The other case was a type 2 diabetic with an unknown cause of death listed. The autopsy case review revealed a plausible suspicion of insulin intoxication by the forensic pathologist, with a history of depression noted, alongside the body presenting with multiple injection marks on the abdomen. This motivated the request for insulin analyses; however, due to sample quality, the analysis could not be verified.

The screening test also identified 3.5% of test cases as potentially hyperglycemia-related. The subsequent cause of death review revealed that seven cases had deaths associated with diabetes mellitus complications, with six of the seven cases having confirmed hyperglycemic VH glucose levels (data were not available for the remaining case). This result is particularly promising for the future application of postmortem metabolomics in aiding the identification of deaths resulting from glycemic disturbances, more so when considering the models used in this study were tailored towards hypoglycemia, based on insulin intoxications, and not hyperglycemia only utilizing a matched number of diabetic coma cases from a greater pool of available cases. Though methods are in place for the determination of hyperglycemia-related death in forensics, including VH glucose measures, postmortem metabolomics may be able to expand on this with future investigation.

### 4.5. Strengths and Limitations of the Postmortem Metabolomics Screening Method

The main purpose of the screening method used in this investigation was to filter out, or “screen”, the random test group of postmortem cases for any cases that may be considered hypoglycemia-related. We believe that, with specific regards to this aim, our screening model was successful in filtering out a discreet number (5.9% of the test group) of potential true-positives. This is a key strength of this screening method, as screening such a discreet number of cases allows for efficient and timely review of additional material related to the cases to further filter out any obvious false-positives—such as in this investigation with two cases having hyperglycemic VH glucose levels, and a number of diagnosed causes of death that should theoretically be independent of hyperglycemia.

Limitations are present with the screening method used. The primary limitation is that this screening model is built using only a limited number of insulin intoxication cases, and the previously discussed limitations regarding this also contribute to the screening method. Of important note, the validation of our multivariate model using confirmed insulin intoxications remains pending due to the lack of additional confirmed cases. Thus, any screening of a previously unknown case as hypo, and with any subsequent review of autopsy reports highlighting a plausible motive for hypoglycemia, we cannot definitively conclude that a hypoglycemia-related death has occurred.

Another key limitation of our screening method is that it is based on a class prediction model. Thus, we need to make it abundantly clear that the results we are presenting do not represent a definitive cause of death classification. Rather, we are screening out potential true-positive cases that require further evaluation of supporting material. This type of class prediction modeling could theoretically be used as a method for cause of death identification; however, this requires much more extensive research. It is clear from this investigation that there is a high degree of false-positive cases being predicted as potentially hypoglycemia-related.

In summary, our screening study does show clear promise in aiding in forensic death investigations in relation to the diagnosis of potential hypoglycemia- and insulin-intoxication-related deaths.

## 5. Conclusions

This study investigated the potential of postmortem metabolomics in identifying a metabolic fingerprint for insulin-intoxication-related deaths that could be useful in aiding forensic death investigations. Insulin intoxications, being hypoglycemia-related deaths, could be successfully discriminated from hyperglycemic and presumed normoglycemic deaths by postmortem metabolomics and multivariate statistical modeling. Metabolite identification and analysis show that acylcarnitines, including a majority of hydroxylated-acylcarnitines, were found at significantly decreased levels in hypoglycemia-related deaths. This acylcarnitine profile may be a potential signature for hypoglycemia-related deaths. A screening application was performed by applying this hypoglycemic metabolite fingerprint to a class prediction model, and used to screen of a large random selection of autopsy cases. This screening resulted in a discreet number of random cases being screened as potentially hypoglycemia-related deaths, in which on review of autopsy reports revealed two diabetic individuals with a known and a suspicious cause of hypoglycemia prior death, respectively. Further studies are required to validate the identified metabolic fingerprint in more confirmed insulin intoxication cases. However, this study clearly demonstrates the promise of postmortem metabolomics in aiding forensic death investigations for the identification of hypoglycemic-related deaths, including insulin intoxications.

## Figures and Tables

**Table 1:** Case characteristics for the study groups included in the main multivariate model building.

	Hypo (*n* = 19)	Hyper (*n* = 19)	Control (*n* = 38)
Sex (male/female)	11/8	11/8	22/16
Age (years)	56 (36–70)	59 (47–64)	59 (37–71)
BMI (kg/m^2^)	24.7 (22.1–28.5)	23.4 (22.3–28.7)	24.3 (21.8–28.7)
PMI (days)	6 (4–10)	6 (4–10)	6 (4–9)
* VH glucose (mmol/L)	0.3 (0.1–0.5) †	38.1 (19.4–47.7)	n/a

Data presented as median and quartile range (Q1–Q3). * Vitreous humor (VH) glucose measurements are only reliable for assessing hyperglycemia >10 mmol/L. This measurement is not suitable for confirming a hypoglycemic state, data presented for the hypo group is only to be interpreted that no case included in this group was hyperglycemic. † *n* = 17.

**Table 2:** Demographic overview of the test group used for the classification screening.

	Test Group (*n* = 776)
Sex (male/female)	390/386
Age (years)	61 (45–71)
BMI (kg/m^2^)	25.0 (22.5–29.7)
PMI (days)	7 (5–11)

Data presented as median and quartile range (Q1–Q3). BMI—body mass index; PMI—postmortem interval.

**Table 3:** Identified metabolites discriminating the hypo group from the hyper and control groups.

Identifier	Metabolite	Chain Length *	Mean m/z	Exact m/z	Δ ppm	Hypo/Control		Hypo/Hyper	
						%	*p*-Value **	%	*p*-Value **
*Direct Parent: Acylcarnitines*									
M230T152	Butenylcarnitine	C4:1	230.139	230.1387	1.3	0.77	0.381	0.07	**0.003**
M248T124	Hydroxybutyrylcarnitine	C4-OH	248.149	248.1492	−0.8	0.34	0.151	0.08	**0.014**
M244T198	Tiglylcarnitine	C5:1M	244.154	244.1543	−1.2	0.63	0.065	0.31	**0.004**
M275T335	Heptanoylcarnitine	C7	274.200	274.2013	−4.7	0.63	0.116	0.55	0.062
M305T224	Hydroxyoctanoyl carnitine	C8-OH	304.211	304.2118	−2.6	0.79	0.394	0.21	**0.005**
M344T572	Dodecanoylcarnitine	C12	344.279	344.2795	−1.5	0.32	0.053	0.34	**0.024**
M360T500	Hydroxydodecanoyl carnitine	C12-OH	360.274	360.2744	−1.1	0.62	0.158	0.35	**0.015**
M388T570	Hydroxytetradecanoylcarnitine	C14-OH	388.305	388.3057	−1.8	0.69	0.152	0.38	**0.003**
M386T556	Hydroxytetradecenoylcarnitine	C14:1-OH	386.290	386.2901	−0.3	0.73	0.345	0.27	**0.010**
M416T608	Hydroxyhexadecanoylcarnitine	C16-OH	416.337	416.3371	−0.2	0.62	0.122	0.46	**0.022**
M414T587	Hydroxyhexadecenoylcarnitine	C16:1-OH	414.321	414.3214	−1.0	0.46	0.169	0.34	**0.003**
M412T567	Hydroxyhexadecadienoylcarnitine	C16:2-OH	412.305	412.3057	−1.7	0.63	0.119	0.47	**0.005**
M442T619	Hydroxyoctadecenoylcarnitine	C18:1-OH	442.353	442.3527	0.7	0.69	0.280	0.42	**0.022**
*Other*									
M166T126	7-Methylguanine		166.073	166.0723	4.2	1.64	0.142	2.13	**0.022**
M283T132	1-Methylinosine		283.103	283.1037	−2.5	1.67	0.175	1.78	0.089
M303T133	Histidylphenylalanine		303.145	303.1452	−0.7	0.73	0.545	0.25	0.092
M209T138	5-Hydroxyindoleacetic acid		192.066	192.0655	2.6	0.64	0.142	0.52	0.121
M234T374	3,5-Dihydroxyphenylvaleric acid		211.096	211.0965	−2.4	1.05	0.831	1.92	**0.004**
M382T599	Sphinganine 1-phosphate		382.273	382.2717	3.4	1.34	0.211	1.63	**0.036**
M302T620	Sphinganine		302.305	302.3054	−1.3	1.60	0.223	1.85	0.081

* Number of carbons, saturations, and alcohols on the ester group of acylcarnitines; ** FDR-adjusted *p*-values, bold values highlight significant results *p* < 0.05. Identified metabolites were matched according to m/z to the HMDB and/or METLIN public databases, and as such, constitute level 2 or 3 putative metabolite identification according to the MSI guidelines [20].

**Table 4:** Reported causes of death for the test group cases that were screened as possible hypoglycemia-related deaths, using a class prediction model based on the insulin intoxication metabolic fingerprint.

Hypo Prediction, *n* = 46 (5.9%)	
Cause of Death	Number
Cardiovascular-related: Myocardial infarction Enlarged heart Heart failure Coronary atherosclerosis	20 (2.6%)
Pulmonary-related: Bronchopneumonia Emphysema Aspiration pneumonia	8 (1.0%)
Substance overdose/poisoning: Drug overdose/poisoning Alcohol overdose/poisoning	6 (0.8%)
Traumatic head injury	2 (0.3%)
Liver cirrhosis	2 (0.3%)
Acidosis	1 (0.1%)
Starvation	1 (0.1%)
*Undetectable cause of death*	6 (0.8%)

(%) are total percent of test group (*n* = 776) used for screening using a class prediction model.

**Table 5:** Reported causes of death for the test group cases that were screened as possible hyperglycemia-related deaths, using a class prediction model based on the insulin intoxication metabolic fingerprint.

Hyper Prediction, *n* = 26 (3.5%)	
Cause of Death	Number
Substance overdose/poisoning: Drug overdose/poisoning Alcohol overdose/poisoning	8 (1.0%)
Diabetes mellitus-related: Diabetes with ketoacidosis Diabetes with coma	7 (0.9%)
Cardiovascular-related: Enlarged heart Coronary atherosclerosis Cerebral hemorrhage	3 (0.4%)
Hanging	3 (0.4%)
Multiple organ failure	1 (0.1%)
Burns and inhalation of smoke	1 (0.1%)
Ketoacidosis	1 (0.1%)
*Undetectable cause of death*	2 (0.3%)

(%) are total percent of test group (*n* = 776) used for screening using a class prediction model.

## Data Availability

Data sharing is not applicable to this article.

## Supplementary Materials

The following supporting information can be downloaded at: https://www.mdpi.com/2218-1989/13/1/5/s1, File S1: R code for XCMS pre-processing and CAMERA annotation; Table S1: XCMS and CAMERA parameters; Table S2: Positive drug and alcohol use found in postmortem toxicological screening for cases included in hypo, hyper, and control groups. Hyper and control group positivity does not reflect any diagnosed contributing factor to cause of death. Table S3: Unidentified discriminating features associated with the hypo group. Table S4: Reported causes of death for the test group cases that were screened as the control group, of presumed normoglycemic cases, using a class prediction model based on the insulin intoxication metabolic fingerprint. Table S5: Reported causes of death for the test group cases that failed to reach the probability threshold for group screening, using a class prediction model based on the insulin intoxication metabolic fingerprint. Table S6: Reported causes of death for the test group cases that were screened into multiple groups, using a class prediction model based on the insulin intoxication metabolic fingerprint. Figure S1: Multivariate modeling using unsupervised principal component analysis (PCA). (A) PCE score plot plotting the first two principal components, of seven, comparing control (green circles) and hypo (red triangles) groups. (B) PCA score plot plotting the first two principal components, of five, comparing hyper (blue squares) and hypo (red triangles) groups; Figure S2: Multivariate modeling using supervised orthogonal partial least squares-discriminant analysis (OPLS-DA) comparing group separation using only the 85 features found to discriminant the hypo group from control and hyper groups. (A) OPLS-DA score plot comparing control (green circles) and hypo (red triangles) group. (B) OPLS-DA score plot comparing hyper (blue squares) and hypo (red triangles) groups.
