# Supplementary material for: Postmortem Metabolomics of Insulin Intoxications and the Potential Application to Find Hypoglycemia-Related Deaths
Source: Metabolites. 2022 Dec 20;13(1):5. doi: 10.3390/metabo13010005 (PMC9912265; doi:10.3390/metabo13010005)
Supplement: Supplementary file 1 [file metabolites-13-00005-s001.zip › metabolites-2070202-supplementary.pdf]

Supplemental Information

Postmortem metabolomics of insulin intoxications and the potential application to find hypoglycemia-related deaths

Liam J. Ward [1,*], Gustav Engvall [2], Henrik Green [1,3], Fredrik C. Kugelberg [1,3], Carl Söderberg [1,†] and Albert Elmsjö [1,†]

[1] Department of Forensic Genetics and Forensic Toxicology, National Board of Forensic Medicine, 587 58 Linköping, Sweden
[2] Department of Forensic Medicine, National Board of Forensic Medicine, 587 58 Linköping, Sweden
[3] Division of Clinical Chemistry and Pharmacology, Department of Biomedical and Clinical Sciences, Linköping University, 581 83 Linköping, Sweden
[†] Authors contributed equally as senior authors
[*] Correspondence: liam.ward@rmv.se

List of Supplemental Information:

Supplement Appendix S1: R code for XCMS pre-processing and CAMERA annotation

Supplement Table S1: XCMS and CAMERA parameters.

Supplement Table S2: Positive drug and alcohol use found in postmortem toxicological screening for cases included in hypo, hyper, and control groups. Hyper and Control group positivity does not reflect any diagnosed contributing factor to cause of death.

Supplement Table S3: Unidentified discriminating features associated with the hypo group.

Supplement Table S4: Reported causes of death for the test group cases that were screened as the control group, of presumed normoglycemic cases, using a class prediction model based on the insulin intoxication metabolic fingerprint.

Supplement Table S5: Reported causes of death for the test group cases that failed to reach the probability threshold for group screening, using a class prediction model based on the insulin intoxication metabolic fingerprint.

Supplement Table S6: Reported causes of death for the test group cases that were screened into multiple groups, using a class prediction model based on the insulin intoxication metabolic fingerprint.

Supplement Figure S1: Multivariate modeling using unsupervised principle component analysis (PCA). (A) PCA score plot plotting the first two principle components, of seven, comparing control (green circles) and hypo (red triangles) group. (B) PCA score plot plotting the first two principle components, of five, comparing hyper (blue squares) and hypo (red triangles) groups.

Supplement Figure S2: Multivariate modeling using supervised orthogonal partial least squares-discriminant analysis (OPLS-DA) comparing group separation using only the 85 features found to discriminant the hypo group from control and hyper groups. (A) OPLS-DA score plot comparing control (green circles) and hypo (red triangles) group. (B) OPLS-DA score plot comparing hyper (blue squares) and hypo (red triangles) groups.

Supplement Appendix S1: R code for XCMS pre-processing and CAMERA annotation

```
library("xcms")
library("CAMERA")
xset<-xcmsSet(method="centWave",
ppm=30,peakwidth=c(3,20),snthresh=3, noise=1000)
xset<-group(xset, mzwid=0.05,bw=3)
xset2<-retcor(xset,method="obiwarp",response=10,plottype="d")
xset2<-group(xset2, mzwid=0.05,bw=3,minfrac=0.5)
xset3<-fillPeaks(xset2)
an<-xsAnnotate(xset3)
anF<-groupFWHM(an, perfwhm=0.6)
anC<-groupCorr(anF)
anFI<-findIsotopes(anC)
anFA<-findAdducts(anFI, polarity="positive")
write.csv(getPeaklist(anFA), file = "result_table.csv")
```

Supplement Table S1: XCMS and CAMERA parameters

Step	Parameter	Value
xcmsSet	method	centWave
	ppm	30
	peakwidth	3, 20
	snthresh	3
	noise	1000
group - 1	method	peakDensity
	mzwid	0.05
	bw	3
retcor	method	obiwarp
	response	10
	plottype	d
group - 2	method	peakDensity
	mzwid	0.05
	bw	3
	minfrac	0.5
CAMERA	perfwhm	0.6

Supplement Table S2: Positive drug and alcohol use found in postmortem toxicological screening for cases included in hypo, hyper, and control groups. Hyper and Control group positivity does not reflect any diagnosed contributing factor to cause of death.

	**Hypo (n = 19)**	**Hyper (n = 19) ***	**Control (n = 38) ***
Drug positive (any)	74%	63%	66%
• Sleep and anxiety	53%	32%	39%
• Antidepressants	26%	37%	42%
• Opioids	16%	11%	3%
• Amphetamine	11%	5%	3%
Alcohol positive	16%	11%	32%

Frequency (%) of positive drug/alcohol findings from toxicological screening performed. Note, the frequencies included poly-drug usage. Drug positive (any) findings are listed, together with frequencies of four common drug classes found positive in samples.

* in Hyper and Control groups the drug/alcohol usage identified was **<u>NOT</u>** diagnosed as a contributing factor to cause of death by forensic pathologists, and may only reflect therapeutic drug use and/or normal consumption of alcohol.

Supplement Table S3: Unidentified discriminating features associated with the *hypo* group.

Identifier	m/z	rt	Identifier	m/z	rt
M310T112	310.057	112.91	M163T485	163.075	485.58
M194T124	194.118	124.96	M366T546	366.271	546.42
M195T125	195.121	125.67	M343T546	343.293	546.55
M270T127	270.144	127.065	M406T555	406.203	555.255
M334T149	334.185	149.21	M407T555	407.206	555.67
M247T152	247.128	152.76	M59T555	59.0494	555.695
M194T160	194.117	160.155	M457T560	457.335	560.12
M178T164	178.121	164.1	M456T560	456.332	560.2
M208T166	208.133	166.8	M302T563	302.216	563.27
M243T170	243.17	170.29	M464T577	464.337	577.43
M194T172	194.117	172.9	M372T589	372.302	589.88
M337T191	337.036	191.04	M701T591	701.559	591
M130T239	130.208	239.56	M312T602	312.16	602.72
M575T246	575.34	246.45	M747T606	747.593	606.79
M210T251	210.17	251.32	M701T612	701.559	612.06
M302T313	302.196	313.275	M446T613	446.326	613.35
M206T369	206.139	369.59	M720T622	720.567	622.02
M250T371	250.165	371.45	M301T622	301.216	622.24
M489T386	489.315	386.3	M787T629	787.605	629.38
M268T391	268.211	391.87	M788T629	788.615	629.505
M533T399	533.341	399.73	M450T630	450.349	630.39
M296T409	296.206	409.99	M497T633	497.506	633.02
M385T414	385.267	414	M280T642	280.163	642.275
M293T436	293.195	436.4	M734T643	734.569	643.9
M294T436	294.199	436.97	M813T647	813.619	647.71
M195T448	195.122	448.5	M797T655	797.619	655.16
M402T462	402.284	462.56			

Feature unidentified due to lack of matching m/z values to HMDB database able to provide putative identification in accordance to MSI levels 2/3.
m/z – mass-to-charge ratio; rt – retention time (secs).

Supplement Table S4: Reported causes of death for the test group cases that were screened as the control group, of presumed normoglycemic cases, using a class prediction model based on the insulin intoxication metabolic fingerprint.

Control prediction, n = 322 (42.8%)			
**Cause of death**	**Number**	**Cause of death**	**Number**
Cardiovascular-related<br>• Myocardial infarction<br>• Coronary atherosclerosis<br>• Enlarged heart<br>• Cerebral hemorrhage<br>• *Other*	92 (11.9%)	Asphyxia<br>• Hanging<br>• Drowning<br>• Suffocation	57 (7.3%)
Traumatic injury<br>• Gunshot<br>• Skull fracture<br>• Brain stem/spinal cord injury<br>• Internal/external injury<br>• *Other*	54 (7.0%)	Substance overdose/poisoning<br>• Drug overdose/poisoning<br>• Alcohol overdose/poisoning<br>• Carbon monoxide poisoning	48 (6.2%)
Pulmonary-related<br>• Bronchopneumonia<br>• Lobar pneumonia<br>• Aspiration pneumonia<br>• *Other*	14 (1.8%)	Digestive system-related<br>• Gastrointestinal hemorrhage<br>• Gastrointestinal ulcer<br>• Liver cirrhosis<br>• *Other*	11 (1.4%)
Central nervous system-related	6 (0.8%)	Hypothermia	3 (0.4%)
Sepsis	3 (0.4%)	Anaphylactic shock	2 (0.3%)
Neoplasms	2 (0.3%)	Ketoacidosis	1 (0.1%)
Kidney inflammation	1 (0.1%)	Electric shock	1 (0.1%)
Heat exhaustion	1 (0.1%)	Starvation	1 (0.1%)
Diabetes mellitus with coma	1 (0.1%)	***Undetectable cause of death***	22 (2.8%)

(%) are total percent of test group (n = 776) used for screening using a class prediction model

Supplement Table S5: Reported causes of death for the test group cases that failed to reach the probability threshold for group screening, using a class prediction model based on the insulin intoxication metabolic fingerprint.

No class, n = 378 (48.7%)			
**Cause of death**	**Number**	**Cause of death**	**Number**
Cardiovascular-related:<br>• Myocardial infarction<br>• Coronary atherosclerosis<br>• Enlarged heart<br>• Cerebral hemorrhage<br>• *Other*	108 (13.9%)	Substance overdose/poisoning<br>• Drug overdose/poisoning<br>• Alcohol overdose/poisoning<br>• Carbon monoxide poisoning	78 (10.0%)
Traumatic injury:<br>• Gunshot<br>• Skull fracture<br>• Brain stem/spinal cord injury<br>• Internal/external injury<br>• *Other*	59 (7.6%)	Asphyxia<br>• Hanging<br>• Drowning<br>• Suffocation	41 (5.3%)
Pulmonary-related:<br>• Bronchopneumonia<br>• Lobar pneumonia<br>• Aspiration pneumonia<br>• *Other*	22 (2.8%)	Digestive system-related<br>• Gastrointestinal hemorrhage<br>• Gastrointestinal ulcer<br>• Liver cirrhosis<br>• *Other*	13 (1.7%)
Central nervous system-related	9 (1.2%)	Keto-/acidosis	8 (1.0%)
Hypothermia	5 (0.6%)	Diabetes mellitus-related	5 (0.6%)
Sepsis/infection	4 (0.5%)	Neoplasms	2 (0.3%)
Acute renal failure	1 (0.1%)	Anaphylactic shock	1 (0.1%)
Pregnancy-related	1 (0.1%)	***Undetectable cause of death***	21 (2.7%)

(%) are total percent of test group (n = 776) used for screening using a class prediction model

Supplement Table S6: Reported causes of death for the test group cases that were screened into multiple groups, using a class prediction model based on the insulin intoxication metabolic fingerprint.

**Hypo & Control, n = 3 (0.4%)**	
**Cause of death**	**Number**
Cerebral hemorrhage	1 (0.1%)
Drug overdose/poisoning	1 (0.1%)
Gastrointestinal hemorrhage	1 (0.1%)

**Hyper & Control, n = 1 (0.1%)**	
**Cause of death**	**Number**
Cardiac arrest	1 (0.1%)

(%) are total percent of test group (n = 776) used for screening using a class prediction model

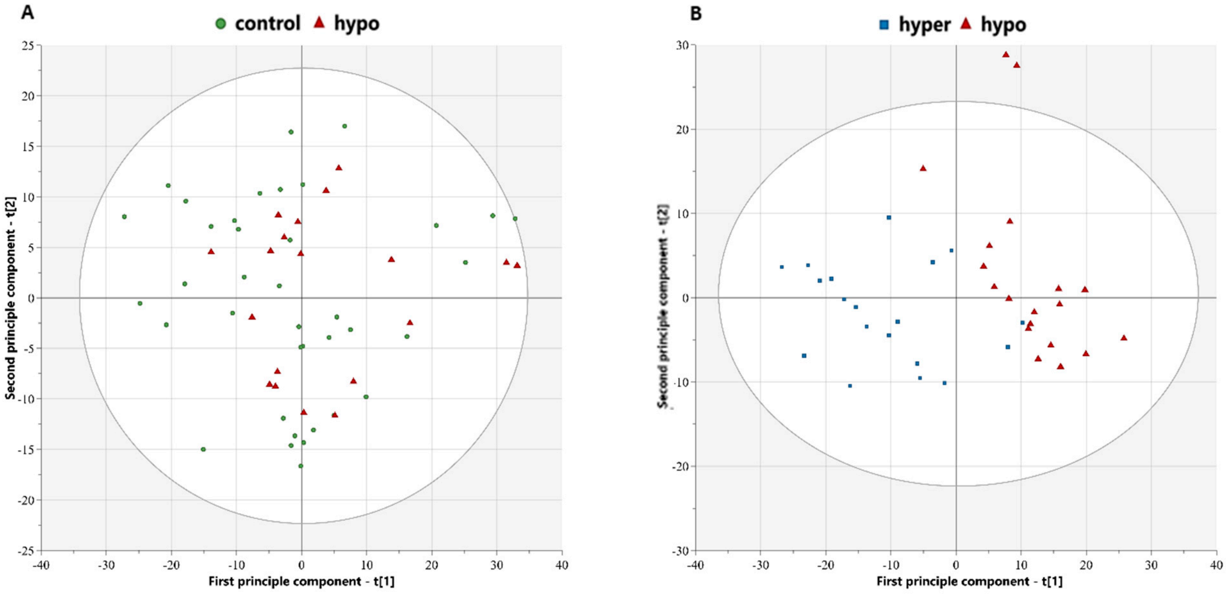

Supplement Figure S1: Multivariate modeling using unsupervised principle component analysis (PCA). (A) PCA score plot plotting the first two principle components, of seven, comparing control (green circles) and hypo (red triangles) group. (B) PCA score plot plotting the first two principle components, of five, comparing hyper (blue squares) and hypo (red triangles) groups.

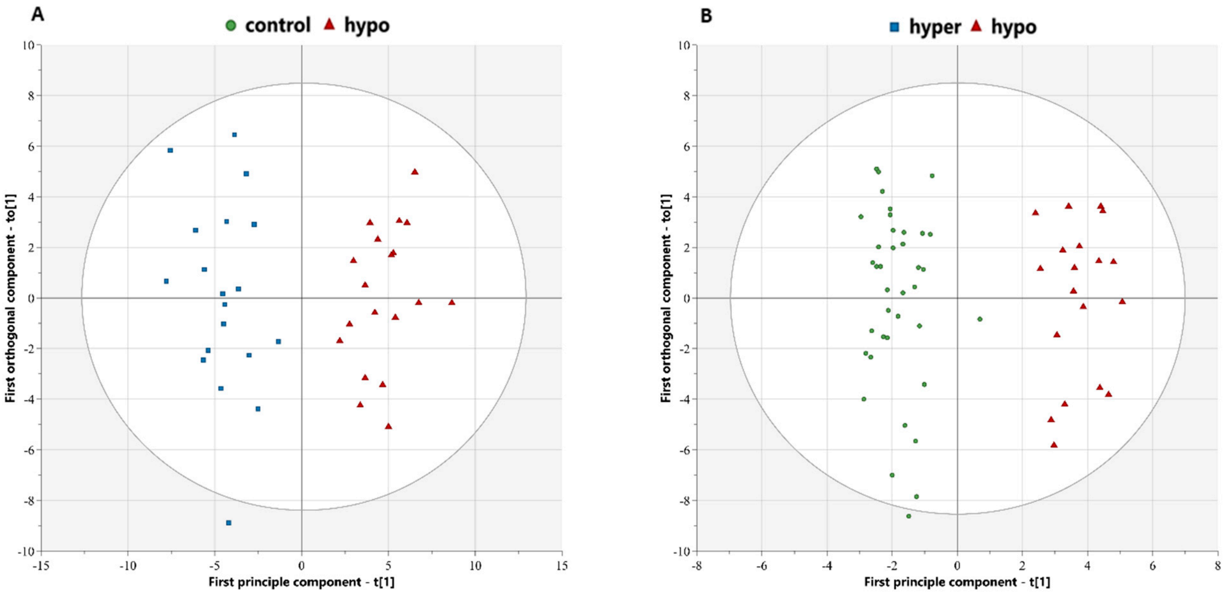

Supplement Figure S2: Multivariate modeling using supervised orthogonal partial least squares-discriminant analysis (OPLS-DA) comparing group separation using only the 85 features found to discriminant the hypo group from control and hyper groups. (A) OPLS-DA score plot comparing control (green circles) and hypo (red triangles) group. (B) OPLS-DA score plot comparing hyper (blue squares) and hypo (red triangles) groups.
